# Calcification of vestibular schwannoma: a case report and literature review

**DOI:** 10.1186/1477-7819-10-207

**Published:** 2012-10-02

**Authors:** Yang Zhang, Jinlu Yu, Limei Qu, Yunqian Li

**Affiliations:** 1 Department of Neurosurgery, First Hospital of Jilin University, 71 Xinmin Avenue, Changchun, 130021, China; 2 Department of Pathology, First Hospital of Jilin University, 71 Xinmin Avenue, Changchun, 130021, China

**Keywords:** Vestibular schwannoma, Calcification, Cerebellopontine angle, Surgery

## Abstract

Calcification rarely occurs in vestibular schwannoma (VS), and only seven cases of calcified VS have been reported in the literature. Here, we report a 48-year-old man with VS, who had a history of progressive left-sided hearing loss for 3 years. Neurological examination revealed that he had left-sided hearing loss and left cerebellar ataxia. Magnetic resonance imaging and computerized tomography angiography showed a mass with calcification in the left cerebellopontine angle (CPA). The tumor was successfully removed via suboccipital craniotomy, and postoperative histopathology showed that the tumor was a schwannoma. We reviewed seven cases of calcified VS that were previously reported in the literature, and we analyzed and summarized the characteristics of these tumors, including the calcification, texture, and blood supply. We conclude that calcification in VS is associated with its texture and blood supply, and these characteristics affect the surgical removal of the tumor.

## Background

Vestibular schwannoma (VS), often called acoustic neuroma, is a common cerebellopontine angle (CPA) tumor. Calcification rarely occurs in vestibular schwannoma, and only seven cases of calcified VS have been reported in the literature [1-6]. Calcification of VS causes difficulties in the differential diagnosis of CPA tumors mainly because calcifications have been found in other CPA tumors, such as meningiomas, cavernous angiomas, gangliogliomas, and solitary fibrous tumors [7-10]. Calcified VS is often misdiagnosed before surgery largely due to insufficient numbers of reported cases and the lack of a comprehensive literature review on this type of tumor. In addition, calcification produces a change in the texture of VS, which can lead to difficulties in surgically removing the tumor [11]. Here, we report a case of calcified VS and summarize a literature review of seven cases of calcified VS. Our aim was to identify the relationship between calcified VS and the texture and blood supply of the tumor in order to guide the surgical treatment of calcified VS.

## Case presentation

**Figure 1:**
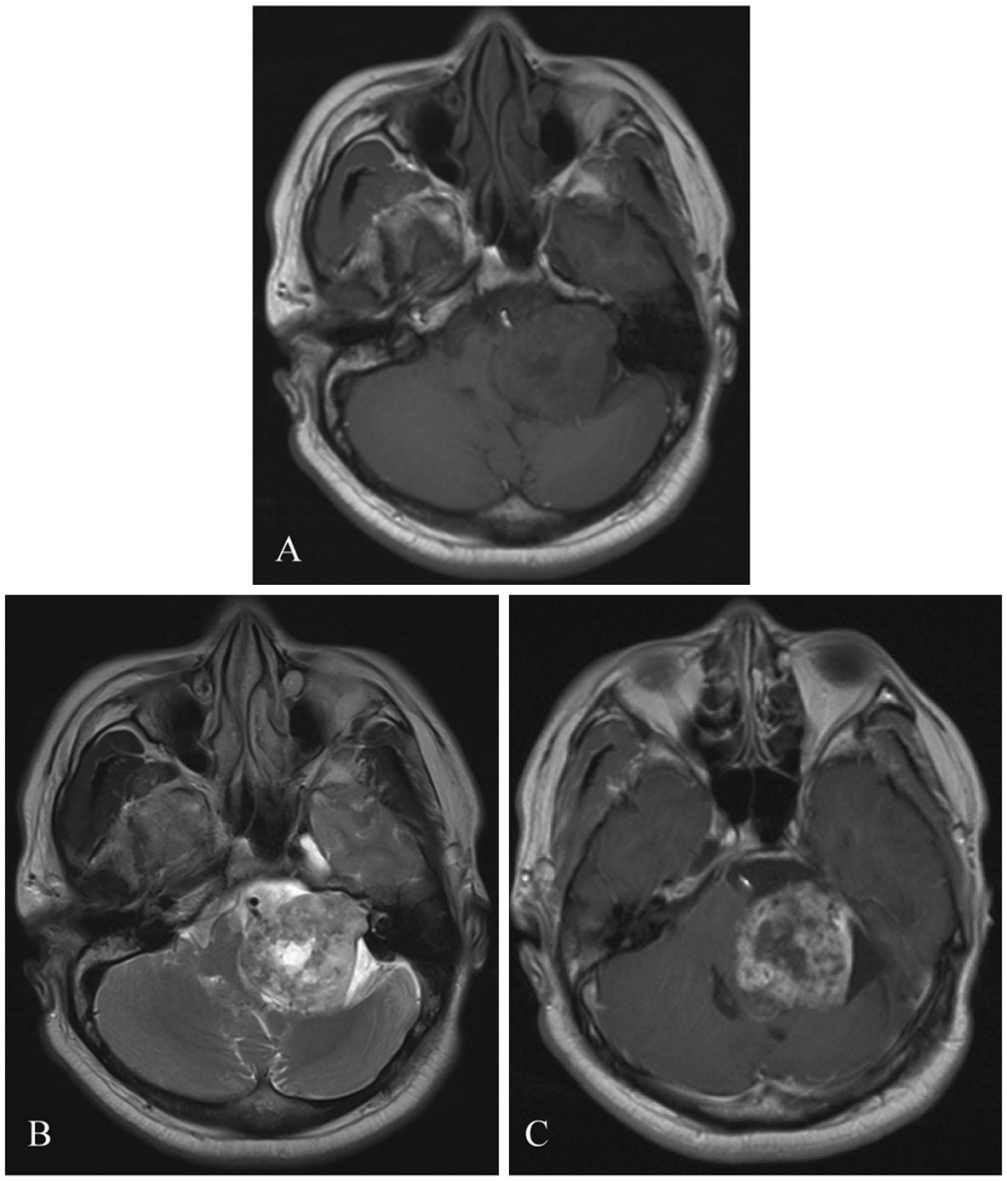
**Preoperative MRI showing a round mass with a size of 5.42 × 4.27 × 5.35 cm in the left CPA region. **(**A**) Hypointensity on the T1-weighted imaging (T1WI). (**B**) Uneven hyperintensity on the T2-weighted imaging (T2WI). (**C**) Heterogeneous enhancement after contrast injection. The left cerebellum, the fourth ventricle, and the brain stem were compressed.

**Figure 2:**
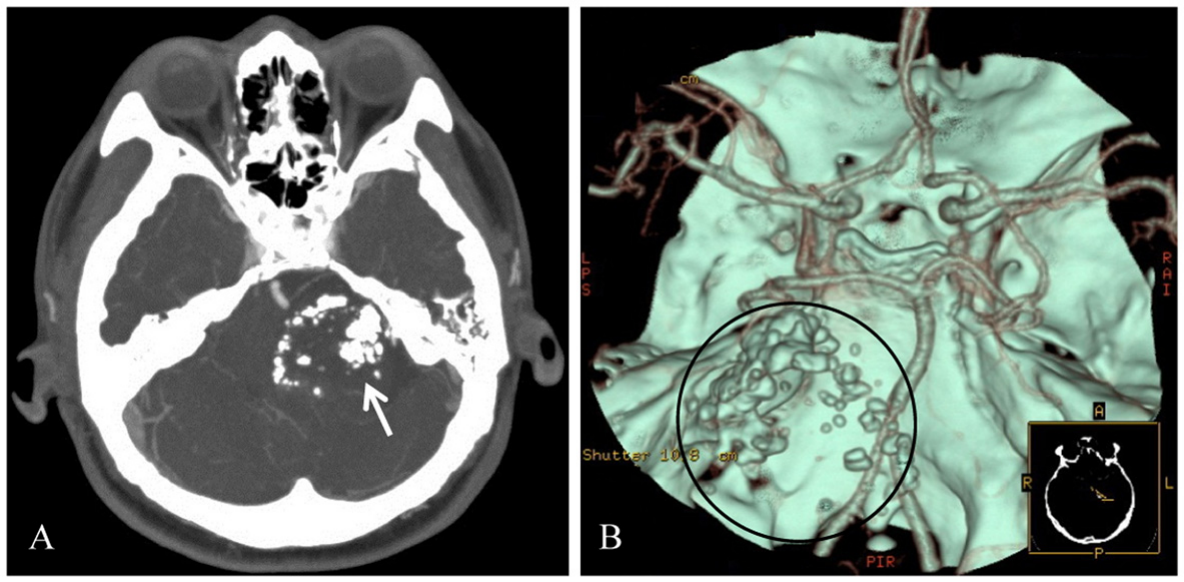
**Preoperative CTA images showing calcification and the intracranial artery. **(**A**) A high-density, patchy calcification shadow on the left CPA region (arrow). (**B**) The tumor (indicated by the circle) has a clear boundary with intracranial vessels. No intracranial artery malformation is observed. The tumor is not stained.

The patient, a 48-year old man, was hospitalized for progressive left-sided hearing loss for 3 years. Upon examination, he had left-sided hearing loss. He also had an abnormal finger-nose pointing test, an abnormal rapid alternating movement, and a heel-knee-shin ataxia on the left side. He did not present with facial palsy and had normal muscle tone in the extremities. Magnetic resonance imaging (MRI) of the brain showed a round mass with a size of 5.42 × 4.27 × 5.35 cm in the left CPA region. The lesion was hypointense in the T1-weighted imaging (T1WI) and unevenly hyperintense in the T2-weighted imaging (T2WI). Heterogeneous enhancement in the tumor was observed in the contrast-enhanced MRI. The left cerebellum, the fourth ventricle, and the brain stem were compressed (Figure 1). Computerized tomography angiography (CTA) of the head revealed a high-density, patchy calcification shadow on the left CPA region. The lesion had a clear boundary with the intracranial vessels, and no intracranial artery malformation was observed. The tumor was not stained on the CTA image (Figure 2). Based on the clinical symptoms and signs, as well as the MRI and CTA findings, the patient was diagnosed with calcified VS.

**Figure 3:**
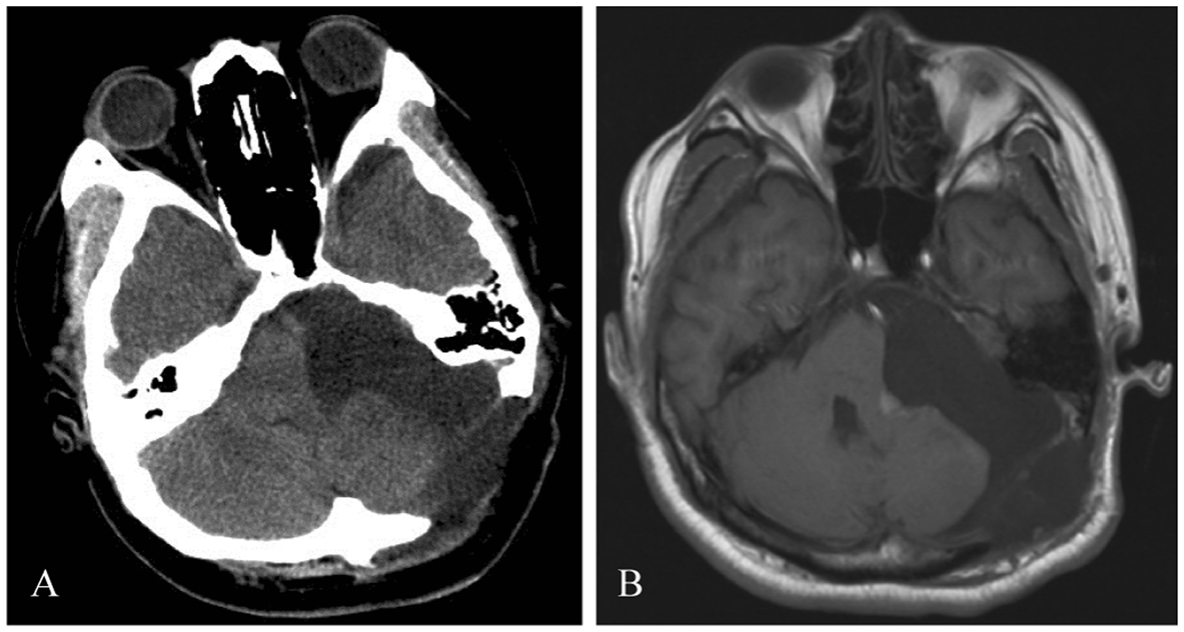
Postoperative CT (A) and MRI (B) showing complete removal of the tumor.

**Figure 4:**
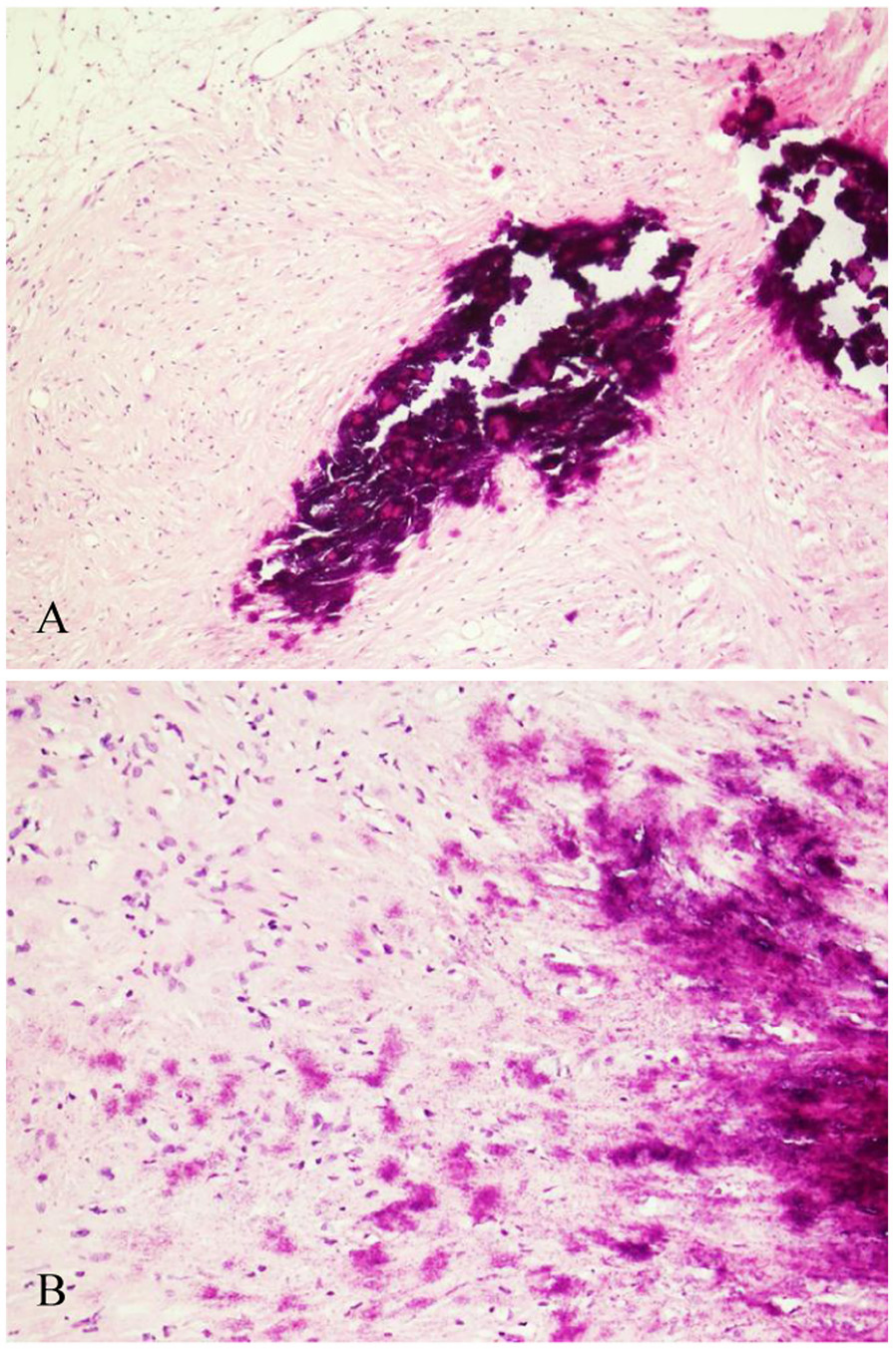
**H&E staining showing strongly stained nuclei and interstitial hyaline degeneration with a large patchy calcification. **(**A**) ×100. (**B**) ×200.

The patient underwent a left retrosigmoid suboccipital craniotomy and total excision of the tumor with preservation of the facial nerve. The tumor, with a complete capsule originating from the internal auditory canal, was highly vascularized. It was yellow-grayish in color, and soft and brittle in texture. The tumor was cystic in the center and calcified in the periphery. After surgery, the patient had mild facial palsy and no improvement in left-sided hearing. At 1 week post surgery, the patient underwent a computerized tomography (CT) scan and MRI, which showed that the tumor was completely removed (Figure 3). The histopathology of the tumor was suggestive of schwannoma. Hematoxylin and eosin (H & E) staining showed strongly stained nuclei and interstitial hyaline degeneration. In addition, a large patchy calcification was observed (Figure 4). The patient had complete loss of left-sided hearing and an improvement in the facial palsy at the 6-month postoperative follow-up appointment.

## Literature review

We performed a Medline literature search to identify cases of calcified VS that had been reported between 1980 and 2011. We found seven cases of calcified VS in six papers. Table 1 shows the summary of the seven cases of calcified VS.

**Table 1:** Summary of calcified VS cases

**Case**	**Author/year**	**Age/sex**	**Duration of disease**	**Symptoms and signs**	**Radiological findings**	**Surgical findings**	**Postoperative complications**
1	[Thomsen/1984]	44/male	2 years	Progressive right-sided hearing loss with a unilateral sensorineural hearing impairment	CT: a mass with a size of 2 × 3 cm with a conglomerate of dense calcification in the right CPA, moderate enhancement after contrast injection, normal internal auditory canal	Approach: translabyrinthine craniotomy twice, subtotal removal of the tumor at the initial operation, and total removal of the tumor after 5 months	Dysdiadokinesis Mild facial palsy
				Examination: spontaneous nystagmus, gait disturbance, and a positive Romberg test		Tumor: whitish, hard, and highly vascularized	
2	[Beskin/1989]	47/male	15 years	Progressive left-sided hearing loss with ringing and itching deep in the left canal	MRI: Hypointensity on T1WI and hyperintensity on T2WI	Approach: left posterior fossa craniotomy, total removal of the tumor after debulking	Mild facial palsy
				Examination: no response in vestibular testing in the left labyrinth	CT: a 3 cm mass with pronounced calcification in the left CPA, enhancement after administration of contrast medium, enlarged internal auditory canal	Tumor: rubbery consistency	
3	[Atlas/1992]	50/male	2 years	Progressive right-sided hearing loss with a unilateral sensorineural hearing impairment	CT: a small mass in the right enlarged internal auditory canal with a conglomerate of dense calcification	Approach: translabyrinthine craniotomy, total removal of the tumor with preservation of the facial nerve	Complete palsy
				Examination: not described		Tumor: adhesion to the facial nerve	
4	[Tosaka/2002]	36/male	30 years	Progressive left-sided hearing loss	MRI: a mass with a size of 3.5 × 3 × 2.5 cm in the CPA, hypointensity on T1WI and hyperintensity on T2WI, heterogeneous enhancement after gadolinium administration	Approach: left suboccipital craniotomy, removal of 90% of the tumor	Hearing was worse on the left side after surgery than before surgery
				Examination: left hearing loss, left canal paresis	CT: significant calcification on the tumor that protruded to the enlarged internal auditory canal	Tumor: whitish, elastic, hard, fibrous, and demarcated with a rich blood supply	No facial palsy
					DSA: no tumor stain		
5	[Katoh/2007]	59/female	15 years	A long history of CPA tumor with no treatment, admitted to the hospital following an epileptic seizure	MRI: a mass with a 3 cm diameter in the CPA, heterogeneous intensity on T1WI and T2WI, heterogeneous enhancement after gadolinium administration	Approach: left suboccipital craniotomy, subtotal removal of the tumor	No facial palsy
				Examination: left deafness, left nystagmus, and left cerebellar ataxia	CT: circular calcification in the periphery of the tumor	Tumor: yellow-grayish, soft, with rich blood supply and old hematoma inside the tumor	
					DSA: no tumor stain		
6	[Gopalakrishnan/2011]	65/male	3 years	Progressive left-sided hearing loss with facial numbness	MRI: hypointensity on T1WI, and heterogeneous hyperintensity on T2WI, heterogeneous enhancement after gadolinium administration	Approach: left suboccipital craniotomy, total removal of the tumor	
				Examination: left hypoesthesia and facial palsy, and sensorineural hearing loss	CT: a mass with a 3 cm diameter in the left CPA with a conglomerate of dense calcification, and enlarged internal auditory canal	Tumor: not described	Death due to myocardial infarction
7	[Gopalakrishnan/2011]	31/female	6 months	Right-sided deafness with facial numbness	MRI: hypointensity on T1WI, and heterogeneous hyperintensity on T2WI, homogenous enhancement after gadolinium administration	Approach: left suboccipital craniotomy, total removal of the tumor	
				Examination: hypoesthesia on the right side of the face, and right sensorineural hearing loss	CT: a mass with a 5 cm diameter in the left CPA with two local deposits of calcification at the periphery of the tumor, and enlarged internal auditory canal	Tumor: not described	Facial palsy (with unknown severity)
8	Present case	48/male	3 years	Progressive left-sided hearing loss	MRI: a mass with a size of 5.42 × 4.27 × 5.35 cm in the left CPA, hypointensity on T1WI, hyperintensity on T2WI, and heterogeneous enhancement in the contrast-enhanced MRI	Approach: left suboccipital craniotomy, total removal of the tumor with preservation of the facial nerve	No improvement in left hearing
				Examination: left hearing loss, left cerebellar ataxia	CTA: a high-density patchy calcification on the left CPA, no intracranial artery malformation, and no tumor stain	Tumor: yellow-grayish in color, soft and brittle in texture with a rich blood supply, and cysts found in the center of the tumor	Mild facial palsy

The seven cases included five men and two women with a mean disease duration of 9.6 years (range, 0.5- to 30 years). All of these patients presented with the initial symptom of hearing loss, and two patients also presented with facial numbness. Neurological examinations were performed in six patients, and CPA symptoms with different severities were identified in these patients.

All the patients underwent CT scanning, and calcification in the VS was clearly observed on the CT scans. A conglomerate of dense calcifications in the VS was reported in three cases, and local calcified deposits were reported in four cases. MRI was performed in five of seven cases. Hypointensity on T1WI and hyperintensity on T2WI were found in four cases, and heterogeneous signals on both T1WI and T2WI were observed in one case. Contrast-enhanced MRI was performed in four of five cases. Heterogeneous enhancement was identified in three cases, and homogeneous enhancement was found in one case. Digital subtraction angiography (DSA) was performed in two cases, in which no tumors were stained in the images.

The tumor was removed via the translabyrinthine approach in two cases and via the suboccipital approach in five cases. Total excision of the tumor was performed in five cases, and subtotal excision was performed in two cases. The detailed surgical procedure was described for four cases, in which the tumor was hard in three cases and cystic and soft in one case. Tumors with a rich blood supply were reported in three cases.

Among the seven cases, two patients had no postoperative facial palsy, two patients had mild postoperative facial palsy, and one patient had complete facial palsy. The severity of postoperative facial palsy was not reported in one case. Postoperative death occurred in one case due to myocardial infarction complications.

## Discussion

It is very rare for VS to demonstrate calcification. When calcification occurs, the schwannoma usually hardens, making it difficult to dissect the tumor surgically [5,12,13]. In addition, VS is located in the CPA at the base of the skull, which restricts surgical access to the tumor. The space constraints make the texture of the tumor an important factor that affects tumor dissection, as with craniopharyngioma in the sellar region [14]. However, it is unclear how calcification, texture, and the blood supply of the calcified VS affect surgical dissection of the tumor. In this study, we presented a case of calcified VS and reviewed the seven reported cases of calcified VS in the literature. We found that calcification in VS is associated with the tumor’s texture and blood supply, and these characteristics affect the surgical removal of the tumor.

The texture of the tumor is one of the factors that affect its surgical removal. The texture is dependent not only on calcification but also on cyst formation and hemorrhage inside the tumors [15,16]. We reviewed seven cases of calcified VS in the literature and found that four cases reported on the tumor texture. In three cases, the tumor was hard, and calcification was found in the VS; however, there was no cystic formation or hemorrhage. In one case, the tumor was soft, and an old hematoma was identified inside the tumor. These findings suggest that intratumoral cyst formation and hemorrhage can soften a calcified VS. Our case agrees with the hypothesis that cysts formed in calcified VS are soft in texture.

The tumor’s blood supply is also a critical factor affecting surgical dissection of the tumor. Three of the seven cases in the literature reported a rich blood supply in the tumors and identified enhancement in contrast-enhanced MRI or CT. However, no tumor staining was identified on angiography in two patients who underwent DSA. Consistent with these reports, no tumor staining was observed with CTA in our case, although a rich blood supply was identified during the operation. The finding that the tumor was not stained in the angiography is likely because the tumor was supplied by a thin artery that was difficult to stain using this procedure [17]. Total excision of the tumor was not performed in two of the seven cases, partly because of the rich blood supply. In our case, a total excision of the tumor was performed because of its softness.

The main surgeries for VS removal are translabyrinthine craniotomy and suboccipital craniotomy [18]. The seven calcified tumors were removed either via the translabyrinthine approach (in two cases) or via the suboccipital approach (in five cases). We used retrosigmoid suboccipital craniotomy to dissect the tumor, since this approach can extensively expose the CPA area and allows for the preservation and reconstruction of cranial nerves [19]. Facial paralysis is the major complication after surgical removal of VS. Postoperative facial palsy occurred in four of the seven cases of calcified VS reported in the literature. Two patients had mild postoperative facial palsy, and one patient had complete facial palsy. The severity of postoperative facial palsy was not reported in one case. In our case, the postoperative facial palsy was mild, and the patient recovered by the postoperative follow-up appointment at 6 months.

A calcified tumor in the CPA suggests a diagnosis of meningioma or cavernous angioma. Identification of the features of these tumors on MRI, CT, and angiography is critical for differential diagnosis among calcified VS, meningioma, and cavernous angioma. Meningiomas usually have a wide base attached to the dura mater and thus have characteristic dural tail signs on the MRI [20]. However, neither our case nor any of the calcified VS cases in the literature had the dural tail sign, suggesting that the dural tail sign may be used to differentiate meningiomas from calcified VS. In addition, meningiomas do not originate from the cranial nerve and, therefore, have different CPA symptoms from VS [21,22]. It is difficult to differentiate calcified VS from calcified cavernous angiomas that originate from the internal auditory canal, since they share some features on MRI (such as isointensity to hyperintensity on T1WI and hyperintensity on T2WI) [23].

## Conclusions

In summary, we report a case of calcified VS and a literature review of seven cases of calcified VS. We analyzed the tumor’s texture and blood supply, the radiological findings, and the surgical procedures. It is difficult to remove hard calcified VS with a rich blood supply. However, cystic formation in VS can soften the tumor, thus facilitating its dissection. We conclude that radiological findings are helpful to differentiate calcified VS from other tumors in the CPA.

## Consent

Written informed consent was obtained from the patient for publication of this case report and the accompanying images. Copies of the written consent are available for review upon request.

## Competing interests

The authors declare that they have no competing interests.

## Authors’ contributions

Yang Zhang and Yunqian Li wrote the initial draft. Jinlu Yu was the surgeon. Limei Qu performed the pathological examination. Yang Zhang and Jinlu Yu contributed equally to this work. All authors read and approved the final manuscript.

### Funding support

This study had no funding support.
